# Discriminating patterns and drivers of multiscale movement in herpetofauna: The dynamic and changing environment of the Mojave desert tortoise

**DOI:** 10.1002/ece3.3235

**Published:** 2017-07-31

**Authors:** Giancarlo Sadoti, Miranda E. Gray, Matthew L. Farnsworth, Brett G. Dickson

**Affiliations:** ^1^ Conservation Science Partners Truckee CA USA; ^2^ Department of Geography University of Nevada Reno NV USA; ^3^ Landscape Conservation Initiative Northern Arizona University Flagstaff AZ USA

**Keywords:** burrows, fencing, *Gopherus agassizii*, movement, solar energy, translocation, weather

## Abstract

Changes to animal movement in response to human‐induced changes to the environment are of growing concern in conservation. Most research on this problem has focused on terrestrial endotherms, but changes to herpetofaunal movement are also of concern given their limited dispersal abilities and specialized thermophysiological requirements. Animals in the desert region of the southwestern United States are faced with environmental alterations driven by development (e.g., solar energy facilities) and climate change. Here, we study the movement ecology of a desert species of conservation concern, the Mojave desert tortoise (*Gopherus agassizii*). We collected weekly encounter locations of marked desert tortoises during the active (nonhibernation) seasons in 2013–2015, and used those data to discriminate movements among activity centers from those within them. We then modeled the probability of movement among activity centers using a suite of covariates describing characteristics of tortoises, natural and anthropogenic landscape features, vegetation, and weather. Multimodel inference indicated greatest support for a model that included individual tortoise characteristics, landscape features, and weather. After controlling for season, date, age, and sex, we found that desert tortoises were more likely to move among activity centers when they were further from minor roads and in the vicinity of barrier fencing; we also found that movement between activity centers was more common during periods of greater rainfall and during periods where cooler temperatures coincided with lower rainfall. Our findings indicate that landscape alterations and climate change both have the potential to impact movements by desert tortoises during the active season. This study provides an important baseline against which we can detect future changes in tortoise movement behavior.

## 1 INTRODUCTION

Understanding patterns and drivers of animal movement is essential for the development of comprehensive conservation and management plans (Johnson, Wiens, Milne, & Crist, 1992; Lima & Zollner, 1996; Morales et al., 2010). Movement can be impeded by natural factors as well as by human‐induced changes to the environment. For instance, the placement of buildings, roads, and fences may restrict natural animal movement processes such as foraging, dispersal, and gene flow (Balkenhol & Waits, 2009; Cattarino, McAlpine, & Rhodes, 2016; Zeller, McGarigal, & Whiteley, 2012). Similarly, climate change may impact animal movements independently or in tandem with landscape change by affecting exposure stress (Doerr et al., 2011), limiting dispersal (Schloss, Nuñez, & Lawler, 2012; Travis et al., 2013), and increasing disease transmission rates (Harvell, Altizer, Cattadori, Harrington, & Weil, 2009).

Numerous methods are available for quantifying animal movement behavior. Genetic tools are frequently applied (e.g., Hagerty, Nussear, Esque, & Tracy, 2011), but they can be limiting because they only reflect movements that result in the production of offspring (e.g., permanent dispersal followed by mating) and they may fail to detect recent changes in movement patterns (Landguth et al., 2010). Direct tracking of individuals—using radio transmitters, for instance—is an alternative approach that enables researchers to detect daily, seasonal, and annual movements at a variety of spatial scales. Beyond identifying the physical location of an animal, these data are increasingly being used to study specific behavioral or activity states (Gurarie et al., 2016; Patterson, Thomas, Wilcox, Ovaskainen, & Matthiopoulos, 2008). For instance, Gurarie et al. (2016) used tracking data from wolves to differentiate dispersive behavior from more localized movement patterns. The ability to identify specific behaviors and study how they respond to environmental variability makes direct tracking a valuable tool for conservation research (Cagnacci et al., 2016).

To date, the vast majority of studies that have applied tracking data to the study of behavioral states have focused on endothermic vertebrates (Frair et al., 2005; Gurarie, Andrews, & Laidre, 2009), with relatively little attention given to herpetofauna (Pittman, Osbourn, & Semlitsch, 2014). This is unfortunate because many reptiles and amphibians have limited dispersal abilities (Colino‐Rabanal & Lizana, 2012) and specialized thermophysiological requirements (Lesbarrères et al., 2014), which may increase their sensitivity to anthropogenic changes (Gibbons et al., 2000; Stuart et al., 2004). Given the importance of movement for population persistence, an improved understanding of fine‐scaled movement and associated resource use patterns in herpetofauna is critical for more effective conservation (McIntyre & Hobbs, 1999).

Here, we apply tracking methods to understand movement behavior in the Mojave desert tortoise (*Gopherus agassizii*; hereafter, desert tortoise), which is found in the desert region of the southwestern United States. The desert tortoise is a federally threatened, semifossorial testudine with activity patterns reflecting variation in temperature and resource availability (Woodbury & Hardy, 1948). The species hibernates during the coldest months and exhibits high fidelity to local areas (Harless, Walde, Delaney, Pater, & Hayes, 2009), but during the active season desert tortoises embark on infrequent, longer‐distance forays, moving between networks of burrows (Berry, 1986; O'Connor, Zimmerman, Ruby, Bulova, & Spotila, 1994; Woodbury & Hardy, 1948). In between these longer‐distance forays, movements tend to be short (<200 m; O'Connor et al., 1994) and concentrated within local areas containing one or more burrows (hereafter, activity centers). Like many desert ectotherms, the desert tortoise persists near its physiological limits (Morafka & Berry, 2002). In addition, the species occupies a region impacted by rapid solar energy development and land use conversion in some areas (Lovich & Ennen, 2011). The individual and synergistic effects of these factors require a greater understanding of the relationship between a changing environment and movement patterns to improve conservation and management of desert tortoise populations (Averill‐Murray, Darst, Field, & Allison, 2012; Field, Tracy, Medica, Marlow, & Corn, 2007).

To help inform conservation and offer an approach with utility to others, we sought to elucidate scales and drivers of movement in the desert tortoise. Our first objective was to develop a method that uses radio‐tracking data to discriminate movements within activity centers from those associated with longer‐distance forays between activity centers (hereafter, activity center movements). The ability to focus on activity center movements was critical to our second and overarching objective: To assess how movements among activity centers are influenced by anthropogenic and natural landscape characteristics, vegetation, and weather projected to shift under scenarios of climate change.

## 2 MATERIALS AND METHODS

### 2.1 Study area and telemetry methods

**Figure 1:**
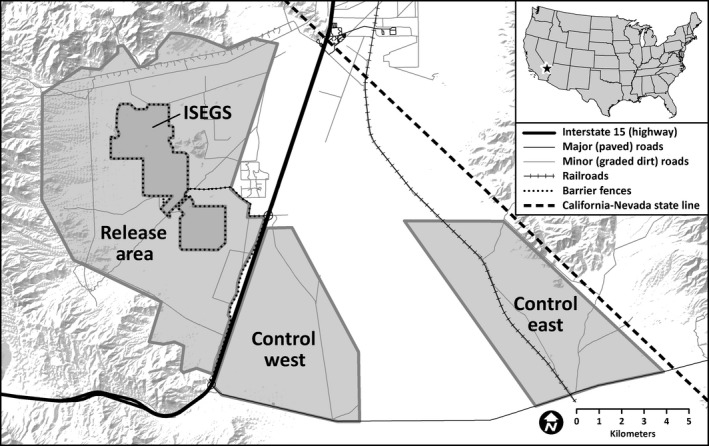
Location of the study area in the Ivanpah Valley of southern California, U.S.A.

Our study area encompasses approximately 18,000 ha and is located in the Ivanpah Valley of southern California, in the eastern Mojave Desert (Figure 1). Most of the area is managed by the U.S. Bureau of Land Management, and at the start of our study contained one operational solar energy facility, the Ivanpah Solar Electric Generating System (ISEGS). The ISEGS facility includes a 1,368‐ha concentrated solar thermal power plant, as well as fences along the boundary that prohibit the passage of tortoises. In addition, the area is bisected by a highway (Interstate 15) and paved public roads accessing ISEGS. Elevation across the valley ranges from 800 to 1,200 m, with topography characterized by alluvial fans and braided washes. The vegetation community is dominated by two perennial shrubs: creosote bush (*Larrea tridentata*) and white bursage (*Ambrosia dumosa*). Precipitation in the region (approximately 10 cm per year) is bimodal with the majority falling as rain in winter and summer (http://www.ncdc.noaa.gov).

Within our study extent, three distinct monitoring areas were established (Figure 1). One area included land immediately adjacent to ISEGS (8,798 ha) and was the release site for a tortoise translocation effort in 2012 (Farnsworth et al., 2015; hereafter, release area). The other two monitoring areas were located in portions of the valley more distant to ISEGS, which were not part of the translocation effort (hereafter, control west [3,560 ha] and control east [4,220 ha]). Although the translocation effort was not a particular focus of this study, we include covariates testing for an effect of translocation treatment on tortoise movement (see covariates below). The three monitoring areas were similar in their vegetation composition and topography—with gently sloping alluvial fans dissected by intermittent or ephemeral streams—but differed to some extent in elevational range (release area = 800–1,200 m, control west = 800–1,000 m, control east = 800–1,100 m), density of minor roads (release area = 0.11 m/ha, control west = 0.28 m/ha, control east = 0.24 m/ha), mean vegetative cover (release area = 23%, control west = 25%, control east = 18%), and the presence of barrier fences (only in the release area). See Farnsworth et al. (2015) for additional description of the study area.

Between October 2010 and October 2015, biologists permitted by the U.S. Fish and Wildlife Service located, captured, and monitored 353 tortoises following standard protocols (USFWS; 2010). All but five tortoises were initially captured in fall 2011. Fifty‐six tortoises initially detected within the ISEGS project footprint were translocated to the release area in 2012 at distances ≤500 m from the location of original capture (Farnsworth et al., 2015). All 353 tortoises were fitted with very high frequency (VHF) radio transmitters (Holohil Systems Ltd., Ontario, Canada). Desert tortoises were located at approximately weekly intervals between 0600 and 1800 during the April–October active season during 2012–2015. Information recorded during encounters included coordinates and a qualitative description of whether tortoises were located inside or outside of burrows. The health and relevant physical dimensions of tortoises were recorded each May and September. Tortoise sex was noted if identifiable. Unknown‐sex individuals were typically subadults. Farnsworth et al. (2015) provide additional information about capture, translocation, and monitoring efforts.

### 2.2 Movement data and estimation

We used a subset of encounter data to quantify tortoise movements. Employing a dataset of desert tortoise encounters collected from 2012 to 2015, a subset was retained to ensure data quality and reduce biases (see Appendix S1 in Supporting Information). First, all encounters were limited to 2013–2015, as this period had consistent quality of environmental predictors (e.g., based on remotely sensed satellite data) and reduced the observed effects of the 2012 translocation event on behavioral patterns (Farnsworth et al., 2015). Second, we restricted our dataset to encounters recorded during May–October (i.e., April data were excluded) because encounters within burrows were more common than those outside burrows in nearly all weeks of this period over 2013–2015. Third, in an effort to focus on activity center movements, we only included data from an encounter at time *t* when the tortoise was in a burrow at time *t* and was also in a burrow at time *t *− 1 (the previous encounter) or time *t *+* *1 (the following encounter). Finally, to control for the length of time between encounters, we retained only those encounters made between five and eight days prior to or following another encounter (see Appendix S1 in Supporting Information for additional information). We assumed weekly re‐encounters adequately captured the temporal scale of continued burrow use or burrow switching (Sah et al., 2016). Using this set of encounters, we calculated straight‐line (Euclidean) distances between encounters for subsequent analyzes.

**Figure 2:**
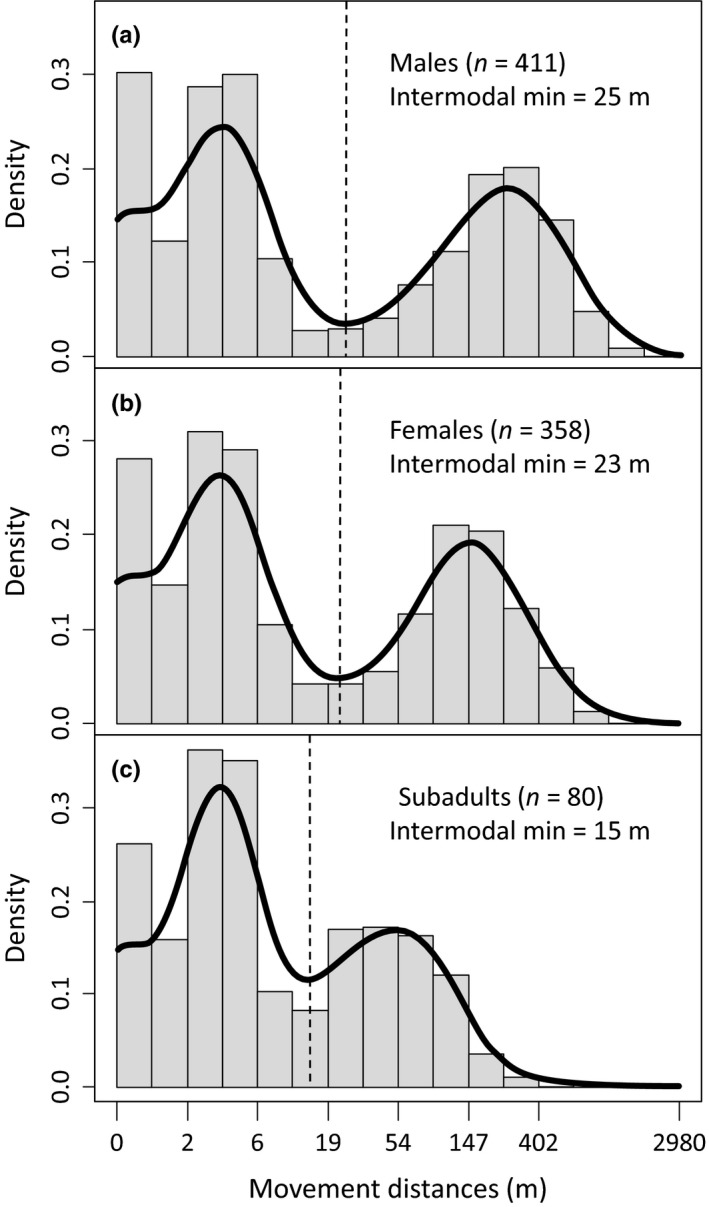
Bimodal distributions of burrow‐to‐burrow movement distances by desert tortoises, based on weekly (5–8 day) radio‐tracking encounters in the Ivanpah Valley, California, U.S.A. Intermodal minima, representing thresholds separating movements within activity centers (below thresholds) from those among activity centers (above thresholds), are shown for (a) males, (b) females, and (c) subadults with dashed vertical lines and were estimate using kernel density estimation. See Appendix S1 for additional information.

Given previous descriptions of spatially clustered movement by desert tortoises (Berry, 1986; O'Connor et al., 1994; Woodbury & Hardy, 1948), we interpreted a bimodal distribution of movement distances as evidence of movement within versus among activity centers. We employed the Silverman test (Silverman, 1981), which uses Gaussian kernel density estimation with bootstrapped bandwidths, to estimate distribution characteristics and to statistically test the null hypothesis of a single distribution mode. We used the local minimum between distribution modes (i.e., the distance at which the kernel density was lowest) as a representative threshold separating movement within and among activity centers. We employed a subset of encounters in testing for bimodality and in determining thresholds (see Appendix S1 for additional information). Threshold values were estimated separately for males, females, and subadults (Figure 2) using the *silvermantest* package (Schwaiger & Holzmann, 2013) in R (R Development Core Team 2014). We treated movement within activity centers versus movement among activity centers as the binary dependent variable (0 and 1, respectively) in our statistical models.

### 2.3 Covariates

We used multiple covariates associated with thermal biology, resource use, reproduction, and landscape permeability to test factors hypothesized to influence activity center movement by tortoises (see below; Table 1). Nonspatial covariates included tortoise sex and age category (Nussear et al., 2012; Ruby et al., 1994), year, and date. Spatial covariates included density of burrows and other individuals (Bulova, 1994; Harless et al., 2009; Sah et al., 2016), the presence of human structures, and shrub cover and topography (Farnsworth et al., 2015). Spatiotemporal covariates included weather and seasonal vegetation characteristics linked to tortoise movements or physiology (Duda, Krzysik, & Freilich, 1999; Franks, Avery, & Spotila, 2011; Henen, Peterson, Wallis, Berry, & Nagy, 1998; Ruby et al., 1994). More details about our calculation methods are provided below. In the event continuous covariates differed strongly among categorical covariates (e.g., winter rainfall among years), we substituted model residuals in the place of covariates. These residuals were derived from ordinary least squares (OLS) models in which the covariate was predicted by the categorical variable.

**Table 1:** Covariates employed in models of activity center movement by desert tortoises in the Ivanpah Valley, California, U.S.A., 2013–2015. All spatial covariates were calculated from values within estimated local use areas. See text and Appendix S1 in Supporting Information for additional covariate information

Abbreviation	Description	Mean	*SD*	Min	Max
Temporal					
Year	Year of observation (%; 2013 = 37.9, 2014 = 32.7, 2015 = 29.4)				
Day	Tortoise encounter Julian day	194	47	94	299
Individual					
Area	Area of observation (%; release = 54.2, control east = 10.0, control west = 35.7)				
Sex	Sex category (%; male = 49.7, female = 42.2, subadult = 8.1)				
Translocated	Translocation status in 2012 (%; resident = 84.3, translocated = 15.7)				
MCL	Midline carapace length (cm)^a^	22.9	42.0	6.7	31.2
Density	Index of local tortoise density (*n*/ha)^a^	0.24	0.43	0	6.2
Landscape					
Slope	Slope of local area (°)	2.2	1.7	0.4	22.3
Roughness	Index of surface roughness (°; *SD* of slope)^a^	1.2	0.3	1.0	3.3
Wash	Area classified as wash (%)	10.4	4.4	0.0	40.2
Minor roads	Presence of maintained dirt roads (%; present = 14.8, absent = 85.2)				
Major roads	Presence of paved roads (%; present = 1.5, absent = 98.5)				
Fencing	Presence of barrier facility fencing (%; present = 11.0, absent = 89.0)				
Burrows	Index of burrow density (*n*/ha)^a^	6.1	7.4	0	67.3
Vegetation					
NDVI_start_	Starting (late March) NDVI^b^	0.11	0.01	0.08	0.19
NDVI_active_	Mean of April‐October NDVI	0.11	0.01	0.08	0.17
NDVI_32_	Mean NDVI in previous 32 days	0.11	0.02	0.07	0.27
NDVI_16_	Mean NDVI in previous 16 days	0.11	0.02	0.07	0.28
Shrub	Area with shrub cover (%)	13.7	5.1	0.1	27.1
Weather					
Tmax_Active_	Mean of April‐October maximum temperature (mm)	31.2	1.1	27.6	32.8
Tmax_32_	Mean maximum temperature in previous 32 days (mm)	32.8	4.0	22.6	39.8
Tmax_16_	Mean maximum temperature in previous 16 days (mm)	33.4	4.3	20.3	41.4
Rain_winter_	Sum of previous November‐March precipitation (mm)^b^	49.8	12.2	31.2	78.8
Rain_active_	Sum of April‐October precipitation (mm)	68.1	9.7	43.8	93.4
Rain_32_	Sum of precipitation in previous 32 days (mm)^c^	9.0	12.6	0.0	69.3
Rain_16_	Sum of precipitation in previous 16 days (mm)^d^	4.2	7.7	0.0	59.7

a As MCL, tortoise density, surface roughness, and burrow density differed strongly among sex category (Kruskal–Wallis χ^2^ ≥ 556.3, *df* = 2, *p *<* *.001), we took residuals from ordinary least squares models of each covariate predicted by sex category for use in models of movement.

b Normalized vegetation difference index. As starting NDVI and winter precipitation differed strongly among years (Kruskal–Wallis χ^2^ ≥ 3,267.6, *df* = 2, *p *<* *.001), we took the residuals from ordinary least squares models of each covariate predicted by sex category for use in models.

c Converted to a categorical covariate; low (<1 mm; 37.9% of observations), medium (≥1, <7.6 mm; 36.6%), and high (≥7.6 mm; 25.5%).

d Converted to a categorical covariate; low (<1 mm; 57.1% of observations), medium (≥1, <4.5 mm; 14.7%), and high (≥4.5 mm; 28.2%).

We used radii of 180 m for males (10.2 ha), 120 m for females (4.5 ha), and 60 m for subadults (1.1 ha) when sampling spatial and spatiotemporal covariates described below. These radii approximate local use areas within which conditions were, we suspect, assessed by tortoises when making decisions to remain within activity centers or move to other activity centers. Radii were estimated from encounters where individuals were returning to a burrow or burrow cluster (see Appendix S1 for additional information). As weekly encounter intervals did not allow for extraction of fine‐scale movement paths between burrows, these circular areas were centered using the geographic midpoint between burrow encounters. The binary category of movement within versus among activity centers associated with this midpoint was linked to the covariates described below.

#### 2.3.1 Temporal covariates

We treated year as a categorical fixed effect in the statistical models described below. We also suspected coarse temporal patterns of movement were related, in part, to patterns of early‐season activity initiation, late‐season activity cessation, and mating (August–October). This seasonal and annual variation was addressed by including the interaction between year and a third‐order (cubic) polynomial of date in all models.

#### 2.3.2 Individual covariates

Individual‐level covariates were monitoring area, sex category (female, male, or subadult), tortoise size index (midline carapace length; MCL), and translocation status (whether or not a tortoise was translocated in 2012). Using methods adapted from Sah et al. (2016), an index of tortoise density was calculated from the number of radio‐marked tortoises nearby in the preceding or following week (see Appendix S1 for additional information).

#### 2.3.3 Landscape covariates

We hypothesized that human infrastructure, topographic variation, and the density of burrows could influence movement in desert tortoises. To address these potential effects, we considered the following variables: the presence of tortoise barrier fencing (surrounding solar facilities and major roads), the presence of roads (paved or graded dirt), an index of wash (intermittent or ephemeral stream) density, the local surface slope, an index of surface roughness, and an index of burrow density. The presence of major (paved) and minor (maintained dirt) roads was calculated from road features in the 2013 TIGER roads dataset (https://www.census.gov/geo/maps-data/data/tiger-line.html, accessed September 20, 2015). Railroads were included in the major road category. These data were evaluated using high‐resolution satellite images (30 m) or aerial photography (1 m) acquired in 2013. Barrier fencing was delineated using this same imagery and based on personal communications with field biologists. Wash density was estimated from high‐resolution aerial photography using robust image classification methods and circuit theory (see Farnsworth et al., 2015). Slope was calculated using 10‐m resolution National Elevation Data (http://nationalmap.gov/elevation.html), while roughness was computed as the standard deviation of slope among 10‐m cells. The approach of Sah et al. (2016) was adapted to address the potential effects of burrow density by including an index calculated from all burrows used during 2012–2015 (following translocation and fence installation; see Appendix S1 for additional information).

#### 2.3.4 Vegetation covariates

Two vegetation measures (shrub density and Normalized Difference Vegetation Index [NDVI]; Pettorelli et al., 2005) were calculated to test their effect on tortoise movements. We suspected shrub density would positively influence probabilities of activity center movement by providing additional thermal refugia. Methods for estimating shrub density are described in Farnsworth et al. (2015). We suspected activity center movements would be more likely during periods and in areas of elevated NDVI, which reflects improved resource availability. This index was calculated from available Landsat 8 surface reflectance scenes in 2013–2015 using Google Earth Engine (https://earthengine.google.com). Landsat 8 has a 30‐m ground resolution and a 16‐day return interval. Data from more or less frequent intervals were available when in overlapping scene edges or areas masked due to clouds, respectively. Mean NDVI was calculated over four temporal periods: (1) the start of spring (late March; an index of winter productivity), (2) the April–October active season, and either (3) the 32‐day or (4) 16‐day period (periods representing one or two Landsat return intervals) immediately preceding the midpoint date between tortoise encounters.

#### 2.3.5 Weather covariates

Daily gridded 4‐km weather data (gridMet; Abatzoglou, 2011), extracted using Google Earth Engine, was used to describe meteorological conditions expected to influence movements by desert tortoises both directly and indirectly. Direct influences included thermal effects (e.g., reduced activity to avoid heat stress), while indirect influences included the effect of rainfall on resource availability (food, surface water) expected to facilitate activity center movement. As with NDVI, total precipitation and mean daily average temperatures were calculated over three temporal windows (active season, the prior 32 days, and the prior 16 days) plus an additional period containing the preceding winter (November–March; Duda et al., 1999).

### 2.4 Statistical models

The probability of activity center movement in desert tortoises was modeled using generalized linear mixed models with a binomial error distribution. All models included nested random effects (intercepts) for individual tortoises within monitoring areas (release area, control west, and control east) and a fixed effect of monitoring year. Additionally, to account for seasonal patterns of activity initiation, breeding, and activity cessation, all models included an interaction between the effect of year and the cubic effect of date. All models included the log‐transformed encounter interval (i.e., 5–8 days) as an offset term.

Two modeling stages were employed to investigate the influence of covariates. In the first stage of modeling, model sets were constructed for each of the four other covariate groups (individual, landscape, vegetation, and weather). Model sets that included covariates having multiple temporal windows (weather and NDVI: season, 32‐days, or 16‐days prior to movement) were built such that only a single temporal window was considered per model. Model sets included first‐order interactions between covariates (e.g., NDVI) and date when we suspected the presence of a seasonally dynamic relationship. All models were fit using the *lme4* package (Bates et al., 2015) in R (R Development Core Team 2014). Model support was evaluated using the Bayesian information criterion (BIC; Schwarz, 1978).

After the most supported (lowest BIC) models among the four covariate group sets were identified in the first modeling stage, a second‐stage (final) candidate set of models (*n *=* *17 models) was built containing (1) a constant, random effect only model, (2) a model containing only temporal (year and date) effects, (2) the four single‐group, BIC‐selected models emerging from the first stage of modeling, and (3) eleven models containing all combinations of the covariates emerging from stage 1 of modeling. If one model in the second‐stage candidate model set had a model weight (*w*_*i*_; calculated from the relative difference in BIC [ΔBIC] between a given model and the model with the lowest BIC) that was <0.95, we employed model averaging (Burnham & Anderson, 2002) across the second‐stage models to calculate parameter estimates associated with each covariate using the *AICcmodavg* package (Mazerolle, 2016). We considered covariates (or their highest‐order terms; e.g., interactions) to be predictive when they had 95% confidence intervals (CIs) that did not include zero. We used the area under the receiver operating characteristic curve (AUC) to assess the ability of models to predict activity center movement. Negligible evidence for spatial or temporal autocorrelation was found (see Appendix S1 for additional information). Variance inflation factors were calculated to investigate collinearity among covariates in models, although no value >3.1 was observed. Pearson chi‐square (χ^2^), Wilcoxon rank‐sum (*W*), and Spearman's rank correlation (ρ) statistics were used to evaluate relationships among covariates.

## 3 RESULTS

We modeled 7,045 movements among 305 tortoises during the active seasons of 2013–2015. On average, we detected 23.1 (*SD* = 8.1) movements per tortoise and 8.2 movements per tortoise per year (*SD* = 3.3). The median distance between sequential encounters was 5.8 m (interquartile range = 1.4–136.4), but the distribution was strongly bimodal for each sex category (Figure 2). Thresholds separating movements within and among activity centers were 25, 23, and 15 m for male, female, and subadult tortoises, respectively. Comparison of models integrating varying combinations of covariate groups indicated greatest support for a model including individual, landscape, and weather effects (Table 2). The AUC of this model (0.72) indicated a good ability to predict activity center movement.

**Table 2:** Final candidate set of models of activity center movement by desert tortoises in the Ivanpah Valley, California, U.S.A., 2013–2015. All models other than the constant model included the interacting effects of year and date. *K* is the number of model parameters, LL is the model log‐likelihood, BIC is the Bayesian information criteria, ΔBIC is the difference between BIC values for each model and that with the lowest BIC, *w*_*i*_ is the model weight, and AUC is the area under the receiver operating curve (a measure of discriminatory ability)

Model	*K*	LL	BIC	ΔBIC	*w*_*i*_	AUC
Individual^a^ + landscape^b^ + weather^c^	29	−4,196.2	8,649.4	0.0	0.97	0.72
Individual + weather	27	−4,208.6	8,656.5	7.1	0.03	0.71
Individual + landscape + vegetation^d^ + weather	33	−4,185.7	8,663.7	14.3	0.00	0.72
Individual + vegetation + weather	31	−4,199.4	8,673.5	24.1	0.00	0.71
Individual + landscape + vegetation	28	−4,245.5	8,739.0	89.6	0.00	0.71
Landscape + weather	21	−4,279.6	8,745.3	95.9	0.00	0.70
Individual + vegetation	26	−4,258.8	8,748.0	98.6	0.00	0.70
Weather	19	−4,291.5	8,751.3	101.9	0.00	0.70
Individual + landscape	24	−4,272.7	8,758.0	108.6	0.00	0.70
Landscape + vegetation + weather	25	−4,269.5	8,760.6	111.2	0.00	0.70
Individual	22	−4,284.0	8,762.9	113.5	0.00	0.70
Vegetation + weather	23	−4,282.8	8,769.3	119.9	0.00	0.70
Landscape + vegetation	20	−4,333.6	8,844.3	194.9	0.00	0.69
Vegetation	18	−4,346.3	8,852.2	202.7	0.00	0.68
Landscape	16	−4,361.3	8,864.3	214.9	0.00	0.68
Year and date	14	−4,372.0	8,868.1	218.7	0.00	0.68
Constant	3	−4,723.0	9,472.6	823.2	0.00	0.51

a Individual and social covariates; includes interactions between date and sex category.

b Topography, structural, and burrow covariates; includes the local presence of minor roads and barrier fences.

c Weather covariates; includes interactions between average daily maximum temperature and total recent rainfall.

d Vegetation covariates; includes interactions between date and recent normalized difference vegetation index.

### 3.1 Temporal and individual effects

**Figure 3:**
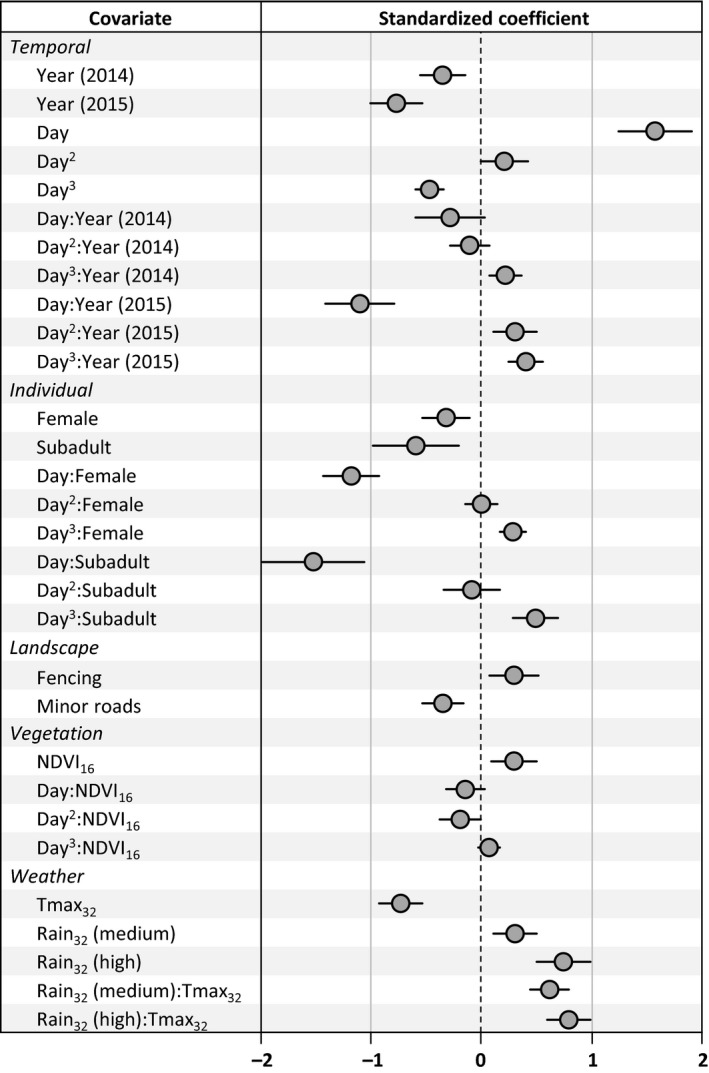
Model‐averaged parameter estimates from second‐stage models of activity center movement by desert tortoises in the Ivanpah Valley, California, U.S.A., 2013–2015. Each parameter estimate is associated with a covariate (shown in Table 1) and indicates the relative effect of the covariate on predicting these two types of movement. Bars indicate 95% confidence intervals, and those not including zero were considered to be predictive covariates. The set of second‐stage models from which values were averaged is provided in Table 2. The intercept (not shown; mean = −2.36, *SE *= 0.14) is associated with 2013 conditions, male tortoises, low rainfall (<1 mm) during the preceding period, and at locations distant from minor roads and barrier fencing. Patterns associated with first‐, second‐, and third‐order polynomials of Julian day (day, day^2^, and day^3^, respectively), as well as their interactions with year and sex category, are illustrated in Figure 4.

**Figure 4:**
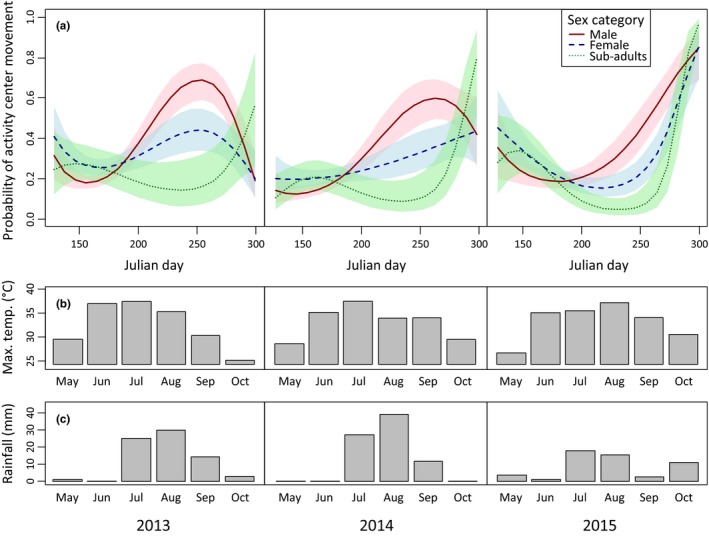
Seasonal changes in the probability of activity center movement by tortoises, as well as seasonal changes in weather in the Ivanpah Valley, California, U.S.A., 2013–2015. Each column represents a different year, and rows indicate (a) variation in tortoise movement by sex category, (b) monthly maximum temperatures (max. temp.), and (c) monthly precipitation totals (rainfall). Shading in panel (a) indicates 95% confidence intervals.

Probabilities of activity center movement varied by year, date, sex category, and their interactions (Figures 3 and 4). Translocation status and tortoise size had a negligible effect on the likelihood of activity center movement in first‐stage modeling and were not selected for second‐stage modeling. Although the probability of activity center movement declined for all desert tortoises during May, males tended to relocate activity centers more often from July through September. Subadults exhibited the lowest probability of activity center movement, particularly through much of July–September. Females exhibited patterns of activity center movement that were intermediate to males and subadults. Although translocation status was not included in second‐stage models, within the release area (*n *=* *3,824 encounters) this covariate exhibited a strong association with fences: 45% of translocated tortoise encounters were near fences, while 23% of resident tortoise encounters were near fences (χ^2^ = 148.5, *df* = 2, *p *<* *.0001). Local tortoise density was not supported for inclusion in second‐stage models.

### 3.2 Landscape effects

**Figure 5:**
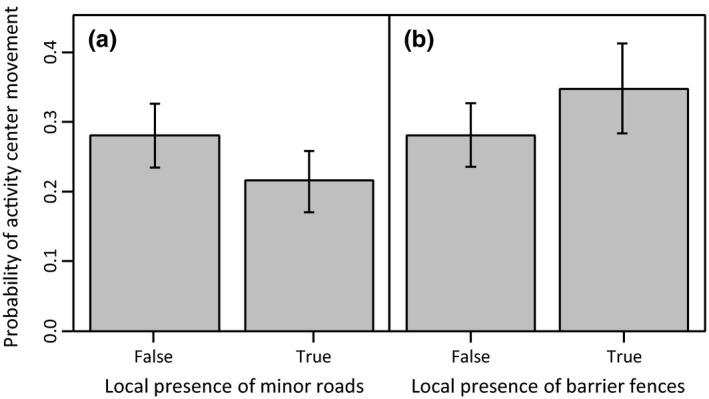
Probability of activity center movement by desert tortoises as predicted by the presence of (a) barrier fences and (b) minor roads within estimated local use areas in the Ivanpah Valley, California, U.S.A., 2013–2015. Shading indicates 95% confidence intervals.

The probability of activity center movement was 31% lower when tortoises were near minor (dirt) roads (0.22) than when they were distant from minor roads (0.28; Figure 5a). In contrast, the probability of activity center movement was 24% higher when tortoises were near barrier fences (0.35) than when they were distant from these features (0.28; Figure 5b). We note that fenced areas also had higher local tortoise densities (*W *=* *1.97 × 10^6^, *p *<* *.0001). Topographic effects (slope and roughness), local burrow density, and wash density did not receive adequate support for inclusion in second‐stage models.

### 3.3 Vegetation effects

**Figure 6:**
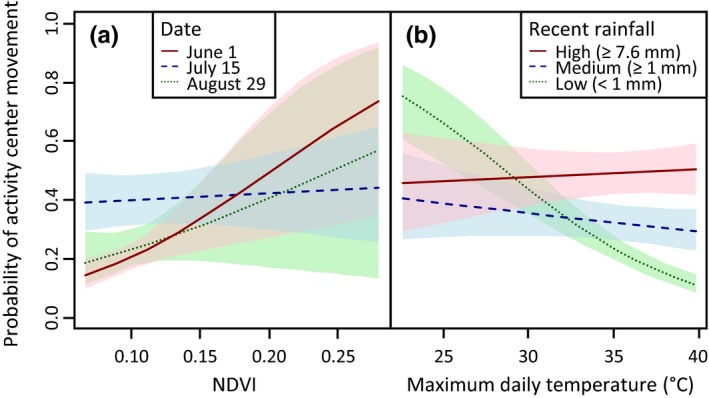
Probability of activity center movement by desert tortoises as predicted by the interaction between (a) normalized difference vegetation index (NDVI) and date and (b) maximum daily temperature and recent rainfall (32‐day mean and total, respectively) in the Ivanpah Valley, California, U.S.A., 2013–2015. Shading indicates 95% confidence intervals.

The best stage 1 model of vegetation effects indicated greater support for using a window of 16 days (vs. 32 days) in calculating NDVI as a covariate (ΔBIC of best 32‐day vs. 16‐day model = 3.7). NDVI had a clear, positive relationship with the probability of activity center movement in the late spring (June 1; Figure 6a), but a weak or highly uncertain relationship at later dates (July 15 and August 29; Figure 6a). Despite these patterns, NDVI contributed little to predicting activity center movement when included in models with topographic and weather covariates (Table 2).

### 3.4 Weather effects

The best stage 1 model indicated greater support for using a 32‐day window versus a 16‐day window in calculating weather covariates (ΔBIC of best 16‐day vs. 32‐day model = 51.8). Our final model provided support for an interaction between temperature and precipitation, such that the probability of activity center movement generally declined with maximum temperature when the preceding period also had low total rainfall (<1 mm; Figures 3 and 6b). This relationship was weak (95% CIs included zero) when rainfall in the previous 32 days totaled over 1 mm.

## 4 DISCUSSION

Movement behavior is important for understanding habitat associations and species distributions (Turchin 1998) and is also critical for developing effective management plans. Here, we studied movement behavior in a reptile of conservation concern, the Mojave desert tortoise—a species that may be vulnerable to environmental alterations stemming from development and climate change. We focused on a particular type of movement: longer‐distance forays among activity centers during the active, nonhibernating season (where each activity center contained a burrow or cluster of burrows). After testing a variety of potential explanatory factors, we found that activity center movements (i.e., movement among activity centers) were best explained by a combination of individual, landscape, and weather variables.

### 4.1 Temporal and individual effects

Variation in activity center movement across sex and age categories followed expected seasonal patterns due to mating and social hierarchies (Bulova, 1997; Harless et al., 2009; Sah et al., 2016). Male desert tortoises, in general, had higher probabilities of activity center movement, and previous work has shown that they have larger home ranges (Harless et al., 2009) and higher rates of burrow switching (Sah et al., 2016) relative to females and subadults. For both males and females, activity center movements tended to be more frequent later in the active season, which coincides with the August–October mating period for desert tortoises.

### 4.2 Landscape effects

Although road‐crossing behavior of herpetofauna is poorly understood, we expected lower probabilities of activity center movement in the vicinity of major roads because many reptile and amphibian species tend to reduce movement when encountering motor vehicles (Andrews, Gibbons, & Jochimsen, 2008). Instead, we detected a lower probability of activity center movement only near minor roads. We suspect that this result may reflect the age structure of tortoise populations near minor roads or correlated characteristics of the landscape. Subadults, which typically exhibit shorter movements and smaller home ranges (Farnsworth et al., 2015), were encountered more frequently than expected near minor roads. Local use areas near minor roads were also relatively steep, rough, and had a relatively larger area classified as wash, characteristics associated with reduced predation risks among subadults (Hagerty et al., 2011; Nafus, Esque, Averill‐Murray, Nussear, & Swaisgood, 2016). Additional investigation is needed to improve understanding of the potential influence of minor roads on desert tortoise movement, such as the possibility of concentrating local activity by providing embankments or other structures for burrow creation or increased resource availability (Latch, Boarman, Walde, & Fleischer, 2011). More detailed descriptions of encounter locations by field personnel will help address this need.

We also detected an effect of barrier fences, as activity center movements were higher in the vicinity of these structures. Barrier fences can be effective in reducing mortality due to road traffic, yet require appropriate placement, maintenance, and (in some cases) passages (Baxter‐Gilbert, Riley, Lesbarrères, & Litzgus, 2015; Jaeger & Fahrig, 2004). Although fences excluding tortoises and other species from areas such as solar facilities can be effective for preventing mortality, these features may present conservation challenges over larger scales (Hayward & Kerley, 2009). The relationship we detected among activity center movement and proximity to fences may result from processes observed in tortoises and other taxa along gradients of fragmentation or individual densities. First, more rapid, directed, or long‐distance movement is often associated with lower‐quality habitat (e.g., with higher predation risks) near fragmenting features such as roads or habitat edges (Fahrig, 2007). Second, while there is little evidence of a reduction in desert tortoise habitat quality near barrier fences, the local presence of any barrier to movement potentially limits the space available for use by animals with activity centered on features such as burrows. Whether this potential limitation resulted in reduced habitat quality is unclear. Finally, the translocation of desert tortoises from within ISEGS to areas adjacent to the fenced ISEGS boundary resulted in higher local tortoise densities, which appeared to persist for several years post‐translocation. This may have caused some animals to move in an effort to find a lower density area. Although density dependence is not considered to have a notable influence on desert tortoise fitness (Doak, Kareiva, & Klepetka, 1994), previous work found that density influenced the movement and social structure of the congeneric gopher tortoise (*Gopherus polyphemus*) (Guyer, Johnson, & Hermann, 2012). Future investigations employing before‐after control‐impact study designs may improve our understanding of the effects of translocation, the installation of barrier fences, and other alterations to desert tortoise habitats and distributions.

### 4.3 Weather and vegetation effects

The influence of weather and vegetation covariates corroborated patterns of thermoregulatory or resource‐exploiting behaviors suggested by previous studies (Duda et al., 1999; Henen et al., 1998; Ruby et al., 1994). The interaction between precipitation and temperature indicates precipitation may facilitate activity center movement during hot periods, possibly by increasing vegetation growth (resulting in more shade or food) or surface water availability. Similar processes were likely captured by the interaction between date and NDVI; increasing greenness (approximating vegetation growth) predicted activity center movement during the generally warm but dry early June period, but not later during the active season. Greater vegetation growth may not be necessary to facilitate movement during July and August because those months are generally wet, so desert tortoises may have sufficient plant foods and shade‐providing vegetation even in years with lower NDVI. These results are consistent with the physiology and behavior of desert tortoises. In contrast with species that travel more widely in periods of low resource availability (e.g., Thompson, Smouse, Scofield, & Sork, 2014), the physiological capabilities of desert tortoises allow for limited activity—resulting in smaller home range size—over prolonged periods of water stress and poor food availability (Henen et al., 1998).

Weather and vegetation variables are often strongly correlated in ecological studies, presenting challenges to decomposing their contributions to movement or other behaviors. In our study, the weekly interval of tortoise encounters, the varying accuracy of gridded weather data across intervals of aggregation, and the coarse temporal scale of remotely sensed vegetation data may have further limited the analysis of short‐term environmental effects (e.g., post‐rainstorm water pooling; Medica, Bury, & Luckenbach, 1980). Nevertheless, by employing both a 16‐ and 32‐day period, we found weather effects were most predictive when considered over an approximately monthly scale. NDVI, while not present in the best model of movement, was more predictive when calculated over a 16‐day period, possibly reflecting more direct, shorter‐term effects of food availability.

### 4.4 Future modeling considerations

Our study was primarily focused on understanding the potential impacts of anthropogenic and climate factors on tortoise movement, and as such our analyses did not address all possible covariates. Future investigations may benefit from considering a greater range of social and environmental characteristics, such as spatial variation in the biochemical composition and digestibility of vegetation (Abella & Berry, 2016; Nagy & Medica, 1986), more detailed measures of local tortoise or burrow density (Nussear & Tracy, 2007), or covariates calculated across multiple spatial extents surrounding encounters. Additionally, the development of models that can address complex movement patterns contained in observations outside of burrows may allow for more integrated investigations of the factors driving movement in this and other herpetofauna.

### 4.5 Conservation implications

Mitigating the impacts of a warming climate will require large‐scale renewable energy development, raising concerns about impacts on biodiversity (Allison, Root, & Frumhoff, 2014). Across the western U.S., federal and state agencies are developing broad‐scale resource management plans, such as the Desert Renewable Energy Conservation Plan (http://www.drecp.org, accessed September 28, 2016), that seek to balance development on public lands with habitat and species preservation. Indeed, the Mojave Desert of southern California is expected to experience significant land conversion with the addition of new energy infrastructure (Lovich & Ennen, 2011; Northrup & Wittemyer, 2013). Approaches to forecasting the potential effects of future development have been applied to other sensitive species in the region, such as the Mohave Ground Squirrel (*Xerospermophilus mohavensis*; Dilts et al., 2016). Similar approaches for the desert tortoise may be possible by integrating information from intensive studies of, for example, space use (e.g., Farnsworth et al., 2015) and genetic connectivity (e.g., Hagerty et al., 2011), with relationships between short‐term movement and human infrastructure.

Daily mean temperatures of the Mojave Desert are projected to increase approximately 2–3°C in the 21st century (Bachelet, Ferschweiler, Sheehan, & Strittholt, 2016). Climate models show poorer agreement in projecting precipitation, but tend to project either wetter winters (January–February) or summers (July–August; Rupp, Abatzoglou, Hegewisch, & Mote, 2013). In contrast to previous investigations (Duda et al., 1999; Franks et al., 2011), we did not identify winter precipitation as a factor influencing active season movement. Below‐normal winter precipitation and above‐normal summer precipitation in our study years may have contributed to this result. While hotter spring (May–June) temperatures are likely to restrict desert tortoise movements, it is unclear whether wetter projected conditions will mitigate hotter temperatures in summer. As individual movement is necessary to maintain connectivity among populations, the potential for future climate conditions to indirectly restrict movement remains a topic of critical interest for this threatened species (Averill‐Murray, Darst, Strout, & Wong, 2013; Barrows, Henen, & Karl, 2016).

The potential population‐level impacts of increased movement resistance due to land cover conversions, road building, and other changes can be considerable (Cushman, 2006). These impacts may be effectively quantified over longer periods and larger areas using genetic approaches (Sinsch, 2014; Van Buskirk, 2012). However, movement is a complex process influenced by factors at multiple spatiotemporal scales; ongoing monitoring (e.g., via radio‐telemetry) allows researchers to more directly investigate how characteristics of the environment influence movement and impact fitness (Pittman et al., 2014). Additionally, information about movement between adjacent and core use (e.g., breeding) areas can be useful in determining areas critical for maintaining populations (Roznik, Johnson, Greenberg, & Tanner, 2009). The negative effects of landscape change on movement may be mitigated, in part, by translocation of individuals (Germano & Bishop, 2009), the placement of crossings (Woltz, Gibbs, & Ducey, 2008), or the (re‐) introduction of disturbances (Greenberg & Waldrop, 2008).

While the effects of projected changes to climate, weather extremes, and weather variability on animal movement are of concern across all vertebrate groups, these changes are of particular concern for herpetofauna given their specialized thermophysiological requirements (Carey & Alexander, 2003). In addition to physiological adaptations, desert tortoises and other herpetofauna have evolved behavioral strategies to tolerate thermal or water stress by either altering their physical environment (e.g., via burrow excavation), migration, or other means. However, burrows and other refuges cannot offer all resources needed for survival. We expect phenological shifts to be one of the most common responses to climate change in herpetofauna, assuming individuals have the capacity to plastically respond to changes in their environment (Carey & Alexander, 2003). Climate change may also indirectly impact animal movement in ways that are similar to landscape change and fragmentation impacts. For example, by potentially altering the availability of focal habitats (e.g., drying of breeding ponds), future climates may impact movement by individuals and, in turn, reduce connectivity within and among populations (Carey & Alexander, 2003). This study provides an important baseline against which we can detect future changes in tortoise movement behavior.

## AUTHORS’ CONTRIBUTIONS

GS, MEG, MLF, and BGD conceived the idea for the manuscript. GS analyzed the data with major contributions from MEG. GS led, and all authors contributed to the writing of the manuscript. MLF and BGD contributed to efforts that produced the field data used in analysis.

## CONFLICT OF INTEREST

None declared.
