# Supplementary material for: Discriminating patterns and drivers of multiscale movement in herpetofauna: The dynamic and changing environment of the Mojave desert tortoise
Source: Ecol Evol. 2017 Jul 31;7(17):7010–22. doi: 10.1002/ece3.3235 (PMC5587480; doi:10.1002/ece3.3235)
Supplement: Supplementary file 1 [file ECE3-7-7010-s001.docx]

# Appendix S1: Supplementary methodological information

Supporting information for

# Discriminating patterns and drivers of multi-scale movement in herpetofauna: the dynamic and changing environment of the Mojave desert tortoise

Giancarlo Sadoti^1,2^*, Miranda E. Gray^1^, Matthew L. Farnsworth^1^, and Brett G. Dickson^1,3^

1. Conservation Science Partners, Truckee, CA 96161, USA
2. Department of Geography, University of Nevada, Reno, NV 89557, USA
3. Landscape Conservation Initiative, Northern Arizona University, Flagstaff, AZ 86011, USA

* Corresponding author: gcsadoti@gmail.com

## Removal of non-burrow encounters

In an effort to focus on movements by desert tortoises within and among activity centers, we conducted a preliminary inspection of distributions (by sex) of movement distances both including and excluding movements originating or ending outside of burrows. Non-movements (distance = 0 m, due to re-encounter of tortoises in the same burrow) were apparent using both samples. However, inclusion of movements originating or ending outside of burrows masked the bimodal distribution within and among activity centers apparent when these non-burrow encounters were excluded. A factor contributing to this pattern may be the frequency of tortoise re-encounters by field personnel (approximately weekly) relative to the frequency of movement outside burrows during the active seasonal period of burrow dependence (typically once or twice daily). The combination of these frequencies is such that apparent between-encounter distances incorporated (1) any movement among activity centers and (2) when outside burrows, any movement within a given day (e.g., to forage) due to factors not recorded by observers such as diurnal temperature variation (Agha et al. 2015).

## Encounter interval

We attempted to control for the potential effects of encounter interval (days between sequential encounters) on patterns of movement (within or among activity centers) by testing their effects in univariate significance tests. We suspected the effect of encounter interval would be evident in tests of correlation (i.e., distances moved by tortoises would be correlated with the length of the encounter interval) or difference (i.e., the distance moved by tortoises would vary across discrete encounter interval categories [in numbers of days]). We tested correlations using Spearman rank correlation tests and variation among encounter interval categories using Kruskal-Wallis tests. These nonparametric tests were employed due to non-normal distributions among movement distances. We included all movement distances over encounters intervals between 0 and 11 days (the 99th percentile of encounter intervals) and all continuous interval ranges (*n* = 66 ranges). For example, if considering an encounter interval range of 2 to 5 days, possible interval lengths would be 0 days (interval range = 2 to 2, 3 to 3, 4 to 4, and 5 to 5), 1 day (interval range = 2 to 3, 3 to 4, and 4 to 5), 2 days (interval range = 2 to 4 and 3 to 5), or 3 days (2 to 5). We considered males, females, and sub-adult individuals separately (Supplementary Table S2 in Appendix S2). We omitted any encounter interval range over which movement distance exhibited a significant correlation with interval length (Spearman *ρ* [rho]; *α* = 0.05) or difference among interval length categories (Kruskall-Wallis *χ*^2^; *α* = 0.05) among males, females, or sub-adults. Among the interval ranges which did not exhibit significant correlations or differences among males, females, or sub-adults (*n* = 7 interval ranges), the interval range of five to eight days had the largest sample of movement distances (*n* = 10,311). Additional restrictions (e.g., date; see main text) further reduced this sample.

## Modes of weekly movement distances

We sought to determine the presence and characteristics of a bimodal pattern of movement distances reflecting movement within and among activity centers. We retained all movements including those of tortoises encountered sequentially in the same burrow. By employing only those encounters within the period of known seasonal activity (per tortoise), we assumed individuals were not torpid and were capable of movement outside of burrows. As we assumed a bimodal distribution among movement distances was common to all tortoises within a season, we first identified a representative minimum number of movements per tortoise that maximized our total sample yet adequately reduced within-tortoise variance in observed movement distances. We first selected 15 as a minimum number of movements for this calculation as it offered an adequate number of unique tortoise-year combinations (n = 37 tortoise-years) in which to test the effect of a decreased number of movements on within-tortoise variance in movement distances. For each number of movements (one through 15), we generated 10,000 bootstrapped samples (with replacement) of movement distances and calculated the means. We then calculated the coefficient of variation (CV) among these 10,000 means for each unique tortoise-year movement number (see Figure S1 in Appendix S2). Finally, using the range of median CV values across each number of movements, we identified the number of movements at which CV was adequately minimized. We used an 80% threshold for this determination. This threshold (0.54) was reached at seven movement distance. The CV at 15 movements was 0.37.

Using all inter-burrow movements from tortoise-years with at least seven movements (*n* = 849 tortoise-years, *n* = 9,026 movements), we then employed kernel density estimation (KDE) to identify the modes within distributions of movement distances. Separately for males (n = 411 tortoise-years), females (n = 358 tortoise-years), and sub-adults (n = 80 tortoise-years), we first tested for bimodality of log-transformed (plus 1) movement distances using the Silverman test ([Silverman, 1981](#_ENREF_6)) with 1,000 bootstrapped samples. Tests for each sex category indicated the null hypothesis of an underlying density of one mode was rejected at *α* = 0.05. This test was conducted using the *silvermantest* package ([Schwaiger & Holzmann, 2013](#_ENREF_5)).

The Silverman test provided estimated bandwidths for females (0.429), males (0.467), and sub-adults (0.403). We used the between-mode local minima as a representative threshold below which movements (per sex category) were identified as movements within activity centers while those above the threshold were identified as movements among activity centers. Thresholds of weekly inter-burrow movement distances, once back-transformed to their original scale, were 23, 25, and 15 m for females, males, and sub-adult tortoises, respectively.

## Estimating area of local use

We assumed tortoises assessed their environment within an area of local use prior to making decision to remain within activity centers or to move among activity centers. Ideally, one would use encounter or other tracking data collected at a temporal resolution that would provide an understanding of daily or sub-daily movement patterns (e.g., area used and distance travelled). Unfortunately, the encounter interval used in this study and most other movement studies of the desert tortoise (approximately weekly) was not frequent enough to explicitly estimate this area. However, the large number of tortoise encounters over the duration of the study—and variation (if small) in the encounter interval—allowed us to approximate this local use area from particular movement sequences.

### Representative sample

The pattern of movements which we assumed would provide insight into the area of local use were those of tortoises encountered outside burrows prior to returning to burrows in (or near; < 10 m) which they were previously encountered. To identify a sample of movements most representative of local burrow-to-resource activities ([Berry, 1986](#_ENREF_1)), we first removed movements of tortoises that were recently translocated (i.e., the release area in 2012). We then retained only those movements from non-burrows (at time *t* - 1) to burrows (time *t*) in which (1) movement was preceded by a movement from a burrow (time *t* - 2) to the non-burrow location (time *t* - 1) and (2) the burrow at *t* - 2 was either the same as at time *t* or within the local movement area. This local area was defined for each sex category by distance thresholds identified for weekly movements above.

As with inter-burrow movement distances, we tested for significant correlation and differences among interval ranges between 0 and 11 days. After removing interval ranges with one or more significant correlation or difference as described above (Supplementary Table S3 in Appendix S2), we deemed movements over shorter intervals (< 7 days) to more reliably describe local patterns and used the range of intervals (0 to 4 days) containing the largest number of movement distances in this group (*n* = 393).

### Radii of estimated local space use

With the sample of movements more reliably approximating local use of areas, we then summarized distances to approximate the radii of these areas for each sex category. We first calculated the median movement distance within each tortoise-year (*n* = 326 tortoise-years). We then calculated the 80th percentile of median distances for each sex category. These distances (and samples) were: females (117 m, *n* = 144), males (176 m, *n* = 144), and sub-adults (54 m, *n* = 38). We rounded these distances to 120, 180, and 60 m to more closely match the resolution (30 m) of Landsat NDVI data employed in modeling. These radii represented areas of 10.2 ha, 4.5 ha, and 1.1 ha, respectively. The full circular area was typically used in extracting covariates (e.g., NDVI) or in calculating individual or burrow density. Exceptions occurred when these areas were situated near barrier fences (surrounding ISEGS, west of Interstate Highway 15 [I-15], and bordering Colosseum Road). In these situations, the area beyond the barrier fence (e.g., within ISEGS) was removed as this area was not available for use by tortoises.

## Covariate calculation

### Individual density

[Sah *et al.* (2016)](#_ENREF_4) calculated local tortoise density within two-month sampling periods using a 100 x 100 m area (1 ha). Values from each day of monitoring were averaged over the sampling period and the sampling area was centered on either individual tortoises or burrows. We modified this approach using a two week period centered on the midpoint day of each (weekly) activity center movement period. We used the estimated local space use area described above for each tortoise dependent on sex-age category.

As an example, given a male encountered at UTM 658710 E and 3929624 N (NAD83, Zone 11) on June 11 and next encountered at UTM 658564 E and 3929540 N on June 17 (midpoint location = 658637 E and 3929582 N, midpoint day = June 14 [a location > 180 m from barrier fencing and thus circular]), we used a window of June 7 to June 21. We first calculated the number of unique tortoises with radio transmitters observed both (1) within 180 m of the midpoint location and (2) encountered between June 7 and June 21. We then divided this number (e.g., 10 tortoises) by the area of the local use area (10.2 ha) for a density index (0.98 tortoises per ha in this example).

### Burrow density

Commercial handheld global positioning system (GPS) units can typically obtain coordinates with a horizontal accuracy of approximately 3 m when receiving a wide area augmentation system (WAAS) signal. We had concerns that this could result in overestimation of the numbers of burrows at locations of repeat burrow encounters. To address this concern, we rounded burrow coordinates to a 3 m grid and removed duplicate burrows. Rather than including only burrows with activity only recently preceding a burrow encounter ([Sah *et al.*, 2016](#_ENREF_4)), we used all 2012–2015 burrows as it was less dependent on sampling date (*r* = -0.02, *n* = 17,273 observations) than active burrows prior to the observation and in the same year (*r* = 0.26). We then divided the total number of burrows within local use areas (180 m, 120 m, or 60 m from movement midpoints for males, females, and sub-adults respectively) by the area of the local use area (10.2 ha, 4.5 ha, or 1.1 ha [or an available section of each area not beyond barrier fencing]). This produced a measure of burrow density that included burrows that were potentially non-active or not yet constructed relative to the tortoise movement in consideration. However, we suspect this estimate of burrow density was likely more reflective of the true active burrow density than estimates derived solely from burrow encounter from a limited sample of radio-marked individuals in a given year.

## Autocorrelation and model accuracy

As non-independence of errors violates statistical assumptions and may bias model estimates, we calculated indices for both spatial and temporal dependence in residuals. Using the most complex model in our final candidate model set, we calculated spatial autocorrelation of model residuals for each weekly time step of the study (*n* = 87 weeks) by constructing Moran’s *I* correlograms with bins of 300-m increments to a maximum distance of two-thirds the maximum distance (19,200 m) between all weekly encounters. We performed 1,000 Monte Carlo permutations on bins containing at least 30 pairs of encounters to generate an empirical distribution from which to assess the probability of observing the given within-bin value of *I* by chance. We used *α* = 0.05 for the first bin, and adjusted this value for other bins ([α divided by bin number; Legendre & Legendre, 1998](#_ENREF_3)). As we detected significant spatial autocorrelation in only 26 (0.6%) of bins after pooling all weeks with an adequate number of encounters (*n =*71 weeks and 4,016 bins), we assumed spatial autocorrelation had a negligible influence on parameter estimates. We employed the *spdep* package ([Bivand, 2013](#_ENREF_2)) for calculation of Moran’s *I*.

We calculated measures of temporal autocorrelation from (weekly) time-lagged residuals for each tortoise from 1 to 20 weeks (within seasons). We used a Spearman rank correlation test (minimum sample = 3 pairs) with an exact calculation of the *P*-value for pairs separately by each lag period. As we found significant temporal autocorrelation in only 68 bins across all 2,528 combined lag periods (2.7% of sample using *n* = 296 tortoises), we assumed adequate temporal independence of errors.

## References

Berry KH (1986) Desert Tortoise (Gopherus agassizii) relocation: Implications of social behavior and movements. Herpetologica*,* **42**, 113-125.

Bivand R (2013) spdep: Spatial dependence: weighting schemes, statistics and models. R package version 0.5-56.

Legendre P, Legendre L (1998) *Numerical ecology,* Elsevier, Amsterdam.

Sah P, Nussear KE, Esque TC, Aiello CM, Hudson PJ, Bansal S (2016) Inferring social structure and its drivers from refuge use in the desert tortoise, a relatively solitary species. Behavioral Ecology and Sociobiology*,* **70**, 1277-1289.

Schwaiger F, Holzmann H (2013) silvermantest: Package which implements the Silverman test. R package version 1.0.

Silverman BW (1981) Using kernel density estimates to investigate multimodality. Journal of the Royal Statistical Society. Series B (Methodological)*,* **43**, 97-99.
