# Supplementary material for: Discriminating patterns and drivers of multiscale movement in herpetofauna: The dynamic and changing environment of the Mojave desert tortoise
Source: Ecol Evol. 2017 Jul 31;7(17):7010–22. doi: 10.1002/ece3.3235 (PMC5587480; doi:10.1002/ece3.3235)
Supplement: Supplementary file 2 [file ECE3-7-7010-s002.docx]

# Appendix S2: Supplementary tables and figures

Supporting information for

# Discriminating patterns and drivers of multi-scale movement in herpetofauna: the dynamic and changing environment of the Mojave desert tortoise

Giancarlo Sadoti^1,2^*, Miranda E. Gray^1^, Matthew L. Farnsworth^1^, and Brett G. Dickson^1,3^

1. Conservation Science Partners, Truckee, CA 96161, USA
2. Department of Geography, University of Nevada, Reno, NV 89557, USA
3. Landscape Conservation Initiative, Northern Arizona University, Flagstaff, AZ 86011, USA

* Corresponding author: gcsadoti@gmail.com

**Table S1.** Numbers of tortoises (and movements) employed in models discriminating movement within and among activity centers in each year, monitoring area, and whether translocated in 2012.

|  | Release | |  |  |  |
| --- | --- | --- | --- | --- | --- |
| Year | Resident | Translocated | Control East | Control West | Total |
| 2013 | 119 (1097) | 48 (370) | 31 (261) | 105 (938) | 303 (2666) |
| 2014 | 111 (848) | 44 (318) | 28 (248) | 101 (889) | 284 (2303) |
| 2015 | 106 (822) | 43 (369) | 24 (193) | 95 (692) | 268 (2076) |

**Table S2.** Sample sizes (*n*), Spearman rank correlations (*ρ*), and Kruskal-Wallis chi-square test values (*χ*^2^) of tortoise movements associated with encounter intervals (numbers of days between encounters). Each row represents a different range of interval values from 0 days (tortoise encountered more than once in a day) to 11 days (the 99th percentile of encounter intervals). The interval range with the largest sample (total *n*) among those ranges without any significant values of *ρ* or *χ*^2^ (*α* = 0.05, shown in bold) was 5 to 8 days (in italics).

| Interval range  (days) | |  | Males | | | Females | | | Sub-adults | | |
| --- | --- | --- | --- | --- | --- | --- | --- | --- | --- | --- | --- |
| Start | End | Total *n* | *n* | *ρ* | *χ*^2^ | *n* | *ρ* | *χ*^2^ | *n* | *ρ* | *χ*^2^ |
| 0 | 1 | 897 | 374 | **0.144** | **7.7** | 333 | 0.084 | 2.3 | 190 | 0.095 | 1.7 |
| 0 | 2 | 1,131 | 495 | **0.093** | **8.5** | 416 | 0.040 | 2.4 | 220 | **0.152** | 5.1 |
| 0 | 3 | 1,589 | 734 | **0.135** | **17.6** | 589 | **0.091** | 6.6 | 266 | 0.084 | 5.0 |
| 0 | 4 | 2,502 | 1,169 | **0.155** | **33.5** | 974 | 0.055 | 7.0 | 359 | 0.044 | 4.8 |
| 0 | 5 | 4,038 | 1,910 | **0.114** | **40.6** | 1,616 | **0.127** | **30.4** | 512 | 0.068 | 6.2 |
| 0 | 6 | 6,697 | 3,210 | **0.076** | **45.6** | 2,711 | **0.070** | **32.7** | 776 | 0.007 | 6.6 |
| 0 | 7 | 10,813 | 5,217 | **0.058** | **50.3** | 4,454 | **0.041** | **32.9** | 1,142 | -0.025 | 7.3 |
| 0 | 8 | 12,813 | 6,206 | **0.041** | **49.8** | 5,268 | **0.058** | **43.4** | 1,339 | 0.014 | 10.3 |
| 0 | 9 | 13,950 | 6,772 | **0.045** | **52.4** | 5,736 | **0.071** | **55.6** | 1,442 | -0.007 | 12.5 |
| 0 | 10 | 14,563 | 7,049 | **0.057** | **67.2** | 6,017 | **0.091** | **85.1** | 1,497 | 0.005 | 13.9 |
| 0 | 11 | 14,764 | 7,157 | **0.065** | **82.1** | 6,095 | **0.097** | **97.9** | 1,512 | 0.012 | 16.8 |
| 1 | 2 | 1,022 | 440 | 0.012 | 0.1 | 373 | -0.014 | 0.1 | 209 | 0.122 | 3.1 |
| 1 | 3 | 1,480 | 679 | **0.086** | 5.7 | 546 | 0.063 | 3.1 | 255 | 0.054 | 3.1 |
| 1 | 4 | 2,393 | 1,114 | **0.123** | **17.6** | 931 | 0.036 | 3.3 | 348 | 0.022 | 2.9 |
| 1 | 5 | 3,929 | 1,855 | **0.090** | **22.3** | 1,573 | **0.115** | **24.1** | 501 | 0.054 | 4.0 |
| 1 | 6 | 6,588 | 3,155 | **0.060** | **25.5** | 2,668 | **0.061** | **25.7** | 765 | -0.003 | 4.7 |
| 1 | 7 | 10,704 | 5,162 | **0.047** | **29.2** | 4,411 | **0.035** | **25.8** | 1,131 | -0.031 | 5.7 |
| 1 | 8 | 12,704 | 6,151 | **0.033** | **28.9** | 5,225 | **0.052** | **35.6** | 1,328 | 0.008 | 8.6 |
| 1 | 9 | 13,841 | 6,717 | **0.037** | **31.3** | 5,693 | **0.066** | **47.4** | 1,431 | -0.012 | 10.9 |
| 1 | 10 | 14,454 | 6,994 | **0.049** | **45.6** | 5,974 | **0.086** | **76.3** | 1,486 | 0.000 | 12.3 |
| 1 | 11 | 14,655 | 7,102 | **0.058** | **60.0** | 6,052 | **0.093** | **88.8** | 1,501 | 0.008 | 15.2 |
| 2 | 3 | 692 | 360 | 0.081 | 2.3 | 256 | 0.078 | 1.5 | 76 | -0.150 | 1.7 |
| 2 | 4 | 1,605 | 795 | **0.088** | **6.9** | 641 | 0.003 | 1.6 | 169 | -0.074 | 2.0 |
| 2 | 5 | 3,141 | 1,536 | 0.044 | 7.4 | 1,283 | **0.099** | **15.4** | 322 | 0.014 | 2.2 |
| 2 | 6 | 5,800 | 2,836 | 0.024 | 7.9 | 2,378 | 0.033 | **15.6** | 586 | -0.044 | 3.9 |
| 2 | 7 | 9,916 | 4,843 | 0.023 | 9.0 | 4,121 | 0.015 | **15.1** | 952 | -0.054 | 5.4 |
| 2 | 8 | 11,916 | 5,832 | 0.012 | 9.2 | 4,935 | **0.036** | **22.6** | 1,149 | -0.004 | 7.8 |
| 2 | 9 | 13,053 | 6,398 | 0.018 | 10.9 | 5,403 | **0.052** | **32.6** | 1,252 | -0.025 | 10.2 |
| 2 | 10 | 13,666 | 6,675 | **0.032** | **23.5** | 5,684 | **0.073** | **58.8** | 1,307 | -0.011 | 11.5 |
| 2 | 11 | 13,867 | 6,783 | **0.041** | **37.0** | 5,762 | **0.080** | **70.6** | 1,322 | -0.002 | 14.3 |
| 3 | 4 | 1,371 | 674 | 0.049 | 1.6 | 558 | -0.034 | 0.6 | 139 | 0.006 | 0.0 |
| 3 | 5 | 2,907 | 1,415 | 0.016 | 1.5 | 1,200 | **0.085** | **11.3** | 292 | 0.058 | 1.0 |
| 3 | 6 | 5,566 | 2,715 | 0.007 | 1.5 | 2,295 | 0.021 | **11.4** | 556 | -0.024 | 2.1 |
| 3 | 7 | 9,682 | 4,722 | 0.013 | 2.2 | 4,038 | 0.007 | **11.0** | 922 | -0.042 | 3.2 |
| 3 | 8 | 11,682 | 5,711 | 0.003 | 2.6 | 4,852 | **0.030** | **17.8** | 1,119 | 0.008 | 5.9 |
| 3 | 9 | 12,819 | 6,277 | 0.011 | 4.0 | 5,320 | **0.046** | **27.3** | 1,222 | -0.015 | 8.2 |
| 3 | 10 | 13,432 | 6,554 | **0.025** | **16.1** | 5,601 | **0.068** | **52.5** | 1,277 | -0.001 | 9.6 |
| 3 | 11 | 13,633 | 6,662 | **0.034** | **29.2** | 5,679 | **0.075** | **64.1** | 1,292 | 0.008 | 12.4 |
| 4 | 5 | 2,449 | 1,176 | -0.010 | 0.1 | 1,027 | **0.102** | **10.7** | 246 | 0.053 | 0.7 |
| 4 | 6 | 5,108 | 2,476 | -0.006 | 0.2 | 2,122 | 0.016 | **10.8** | 510 | -0.036 | 2.0 |
| 4 | 7 | 9,224 | 4,483 | 0.006 | 0.5 | 3,865 | 0.003 | **10.5** | 876 | -0.048 | 3.2 |
| 4 | 8 | 11,224 | 5,472 | -0.003 | 1.0 | 4,679 | 0.028 | **17.0** | 1,073 | 0.006 | 5.9 |
| 4 | 9 | 12,361 | 6,038 | 0.005 | 2.2 | 5,147 | **0.045** | **26.1** | 1,176 | -0.017 | 8.1 |
| 4 | 10 | 12,974 | 6,315 | 0.020 | **13.8** | 5,428 | **0.067** | **50.5** | 1,231 | -0.003 | 9.5 |
| 4 | 11 | 13,175 | 6,423 | **0.030** | **26.6** | 5,506 | **0.074** | **61.9** | 1,246 | 0.006 | 12.3 |
| 5 | 6 | 4,195 | 2,041 | 0.000 | 0.0 | 1,737 | -0.045 | 3.5 | 417 | -0.069 | 2.0 |
| 5 | 7 | 8,311 | 4,048 | 0.010 | 0.4 | 3,480 | -0.024 | 4.1 | 783 | -0.055 | 3.1 |
| 5 | 8 | 10,311 | 5,037 | -0.001 | 0.9 | 4,294 | 0.009 | 8.8 | 980 | 0.006 | 5.8 |
| 5 | 9 | 11,448 | 5,603 | 0.007 | 2.2 | 4,762 | **0.029** | **16.4** | 1,083 | -0.019 | 8.0 |
| 5 | 10 | 12,061 | 5,880 | 0.023 | **13.8** | 5,043 | **0.053** | **38.4** | 1,138 | -0.003 | 9.4 |
| 5 | 11 | 12,262 | 5,988 | **0.034** | **26.4** | 5,121 | **0.061** | **49.1** | 1,153 | 0.007 | 12.2 |
| 6 | 7 | 6,775 | 3,307 | 0.010 | 0.3 | 2,838 | 0.002 | 0.0 | 630 | -0.018 | 0.2 |
| 6 | 8 | 8,775 | 4,296 | -0.003 | 0.9 | 3,652 | **0.034** | **6.5** | 827 | 0.046 | 4.1 |
| 6 | 9 | 9,912 | 4,862 | 0.007 | 2.1 | 4,120 | **0.053** | **15.0** | 930 | 0.012 | 5.8 |
| 6 | 10 | 10,525 | 5,139 | 0.025 | **13.4** | 4,401 | **0.078** | **37.8** | 985 | 0.027 | 7.5 |
| 6 | 11 | 10,726 | 5,247 | **0.037** | **25.8** | 4,479 | **0.087** | **48.6** | 1,000 | 0.039 | 10.5 |
| 7 | 8 | 6,116 | 2,996 | -0.016 | 0.8 | 2,557 | **0.046** | **5.4** | 563 | 0.080 | 3.6 |
| 7 | 9 | 7,253 | 3,562 | 0.001 | 1.8 | 3,025 | **0.063** | **12.0** | 666 | 0.019 | 5.5 |
| 7 | 10 | 7,866 | 3,839 | 0.027 | 12.2 | 3,306 | **0.092** | **31.2** | 721 | 0.038 | 7.0 |
| 7 | 11 | 8,067 | 3,947 | **0.042** | **23.5** | 3,384 | **0.102** | **40.7** | 736 | 0.053 | **9.9** |
| 8 | 9 | 3,137 | 1,555 | 0.032 | 1.6 | 1,282 | 0.031 | 1.3 | 300 | **-0.128** | **4.9** |
| 8 | 10 | 3,750 | 1,832 | **0.072** | **11.4** | 1,563 | **0.076** | **10.3** | 355 | -0.048 | 5.7 |
| 8 | 11 | 3,951 | 1,940 | **0.094** | **21.4** | 1,641 | **0.091** | **16.1** | 370 | -0.014 | **7.9** |
| 9 | 10 | 1,750 | 843 | **0.073** | **4.5** | 749 | 0.070 | 3.7 | 158 | 0.147 | 3.4 |
| 9 | 11 | 1,951 | 951 | **0.103** | **10.4** | 827 | **0.092** | **7.1** | 173 | **0.188** | **6.2** |
| 10 | 11 | 814 | 385 | 0.066 | 1.7 | 359 | 0.055 | 1.1 | 70 | 0.122 | 1.0 |

**Table S3.** Sample sizes (*n*), Spearman rank correlations (*ρ*), and Kruskal-Wallis chi-square test values (*χ*^2^) of movements associated with encounter intervals (numbers of days between encounters). Each observation represents a Euclidean distance between a non-burrow tortoise encounter (encounter *t*) and a burrow in which a tortoises was observed in both encounter *t* – 1 and *t* + 1. Each row in the table represents a different range of between-encounter interval values from 0 days (tortoise encountered more than once in a day) to 11 days (the 99th percentile of encounter intervals). The interval range having both a large sample (total *n*) and overall shorter intervals among those ranges without any significant values of *ρ* or *χ*^2^ (*α* = 0.05, shown in bold) was 0 to 4 days (in italics). NAs result when all movements were within one interval.

| Interval range (days) | |  | Males | | | Females | | | Sub-adults | | |
| --- | --- | --- | --- | --- | --- | --- | --- | --- | --- | --- | --- |
| Start | End | Total *n* | *n* | *ρ* | *χ*^2^ | *n* | *ρ* | *χ*^2^ | *n* | *ρ* | *χ*^2^ |
| 0 | 1 | 125 | 44 | -0.138 | 0.8 | 56 | -0.071 | 0.3 | 25 | NA | NA |
| 0 | 2 | 148 | 56 | -0.040 | 0.9 | 63 | -0.122 | 0.9 | 29 | -0.251 | 1.8 |
| 0 | 3 | 226 | 90 | 0.008 | 0.9 | 102 | 0.089 | 2.4 | 34 | -0.212 | 1.9 |
| 0 | 4 | 393 | 166 | 0.017 | 0.9 | 174 | 0.131 | 4.5 | 53 | 0.156 | 5.3 |
| 0 | 5 | 716 | 338 | **0.182** | **13.4** | 297 | 0.107 | 6.2 | 81 | 0.205 | 6.9 |
| 0 | 6 | 1,254 | 596 | **0.140** | **18.1** | 542 | **0.097** | 9.0 | 116 | 0.011 | 7.9 |
| 0 | 7 | 2,039 | 971 | **0.087** | **19.6** | 898 | **0.095** | 13.0 | 170 | 0.043 | 8.6 |
| 0 | 8 | 2,457 | 1,176 | 0.051 | **19.6** | 1,074 | **0.091** | 14.4 | 207 | 0.063 | 9.2 |
| 0 | 9 | 2,723 | 1,309 | **0.057** | **20.7** | 1,191 | **0.072** | 14.3 | 223 | 0.074 | 9.8 |
| 0 | 10 | 2,868 | 1,382 | 0.035 | **22.6** | 1,255 | **0.068** | 14.4 | 231 | 0.116 | 15.0 |
| 0 | 11 | 2,892 | 1,396 | 0.041 | **24.7** | 1,264 | **0.067** | 14.4 | 232 | 0.113 | 15.1 |
| 1 | 2 | 143 | 53 | 0.029 | 0.0 | 61 | -0.099 | 0.6 | 29 | -0.251 | 1.8 |
| 1 | 3 | 221 | 87 | 0.042 | 0.2 | 100 | 0.106 | 2.2 | 34 | -0.212 | 1.9 |
| 1 | 4 | 388 | 163 | 0.034 | 0.2 | 172 | 0.138 | 4.4 | 53 | 0.156 | 5.3 |
| 1 | 5 | 711 | 335 | **0.193** | **13.0** | 295 | 0.109 | 6.1 | 81 | 0.205 | 6.9 |
| 1 | 6 | 1,249 | 593 | **0.145** | **17.9** | 540 | **0.098** | 9.0 | 116 | 0.011 | 7.9 |
| 1 | 7 | 2,034 | 968 | **0.089** | **19.3** | 896 | **0.095** | **12.9** | 170 | 0.043 | 8.6 |
| 1 | 8 | 2,452 | 1,173 | 0.053 | **19.4** | 1,072 | **0.091** | **14.3** | 207 | 0.063 | 9.2 |
| 1 | 9 | 2,718 | 1,306 | **0.059** | **20.5** | 1,189 | **0.072** | 14.3 | 223 | 0.074 | 9.8 |
| 1 | 10 | 2,863 | 1,379 | 0.037 | **22.4** | 1,253 | **0.068** | 14.4 | 231 | 0.116 | 15.0 |
| 1 | 11 | 2,887 | 1,393 | 0.043 | **24.5** | 1,262 | **0.067** | 14.4 | 232 | 0.113 | 15.1 |
| 2 | 3 | 101 | 46 | 0.026 | 0.0 | 46 | 0.171 | 1.3 | 9 | 0.087 | 0.1 |
| 2 | 4 | 268 | 122 | 0.007 | 0.0 | 118 | 0.093 | 2.0 | 28 | **0.429** | 5.0 |
| 2 | 5 | 591 | 294 | **0.173** | **9.2** | 241 | 0.043 | 2.3 | 56 | 0.180 | 4.9 |
| 2 | 6 | 1,129 | 552 | **0.116** | **12.2** | 486 | 0.050 | 3.2 | 91 | -0.043 | 6.9 |
| 2 | 7 | 1,914 | 927 | **0.065** | **12.9** | 842 | 0.064 | 5.4 | 145 | 0.003 | 7.6 |
| 2 | 8 | 2,332 | 1,132 | 0.031 | **13.2** | 1,018 | **0.064** | 6.3 | 182 | 0.031 | 8.0 |
| 2 | 9 | 2,598 | 1,265 | 0.040 | 14.0 | 1,135 | 0.047 | 6.4 | 198 | 0.044 | 8.4 |
| 2 | 10 | 2,743 | 1,338 | 0.018 | **16.3** | 1,199 | 0.043 | 6.4 | 206 | 0.090 | 13.1 |
| 2 | 11 | 2,767 | 1,352 | 0.025 | **18.3** | 1,208 | 0.043 | 6.4 | 207 | 0.086 | 13.2 |
| 3 | 4 | 245 | 110 | -0.001 | 0.0 | 111 | 0.037 | 0.2 | 24 | 0.333 | 2.6 |
| 3 | 5 | 568 | 282 | **0.168** | **8.3** | 234 | 0.013 | 0.2 | 52 | 0.099 | 2.5 |
| 3 | 6 | 1,106 | 540 | **0.108** | **10.8** | 479 | 0.036 | 0.8 | 87 | -0.112 | 4.9 |
| 3 | 7 | 1,891 | 915 | 0.058 | **11.3** | 835 | 0.055 | 2.7 | 141 | -0.037 | 5.4 |
| 3 | 8 | 2,309 | 1,120 | 0.025 | **11.7** | 1,011 | 0.057 | 3.5 | 178 | 0.001 | 5.7 |
| 3 | 9 | 2,575 | 1,253 | 0.035 | 12.5 | 1,128 | 0.040 | 3.6 | 194 | 0.017 | 6.1 |
| 3 | 10 | 2,720 | 1,326 | 0.013 | **14.8** | 1,192 | 0.037 | 3.6 | 202 | 0.065 | 10.5 |
| 3 | 11 | 2,744 | 1,340 | 0.020 | **16.8** | 1,201 | 0.037 | 3.6 | 203 | 0.061 | 10.7 |
| 4 | 5 | 490 | 248 | **0.160** | **6.3** | 195 | -0.010 | 0.0 | 47 | -0.032 | 0.0 |
| 4 | 6 | 1,028 | 506 | 0.086 | **7.7** | 440 | 0.025 | 0.3 | 82 | -0.194 | 3.3 |
| 4 | 7 | 1,813 | 881 | 0.040 | **7.8** | 796 | 0.048 | 1.9 | 136 | -0.081 | 3.7 |
| 4 | 8 | 2,231 | 1,086 | 0.009 | 8.5 | 972 | 0.051 | 2.7 | 173 | -0.030 | 4.0 |
| 4 | 9 | 2,497 | 1,219 | 0.021 | 9.1 | 1,089 | 0.034 | 2.8 | 189 | -0.011 | 4.3 |
| 4 | 10 | 2,642 | 1,292 | 0.000 | 11.7 | 1,153 | 0.031 | 2.8 | 197 | 0.039 | 8.6 |
| 4 | 11 | 2,666 | 1,306 | 0.007 | 13.6 | 1,162 | 0.031 | 2.8 | 198 | 0.036 | 8.7 |
| 5 | 6 | 861 | 430 | 0.003 | 0.0 | 368 | 0.029 | 0.3 | 63 | -0.184 | 2.1 |
| 5 | 7 | 1,646 | 805 | -0.009 | 0.1 | 724 | 0.046 | 1.6 | 117 | -0.028 | 2.6 |
| 5 | 8 | 2,064 | 1,010 | -0.032 | 1.4 | 900 | 0.048 | 2.2 | 154 | 0.016 | 3.1 |
| 5 | 9 | 2,330 | 1,143 | -0.014 | 1.8 | 1,017 | 0.030 | 2.4 | 170 | 0.034 | 3.4 |
| 5 | 10 | 2,475 | 1,216 | -0.034 | 4.9 | 1,081 | 0.026 | 2.4 | 178 | 0.089 | 8.1 |
| 5 | 11 | 2,499 | 1,230 | -0.026 | 6.7 | 1,090 | 0.026 | 2.4 | 179 | 0.085 | 8.2 |
| 6 | 7 | 1,323 | 633 | -0.013 | 0.1 | 601 | 0.031 | 0.6 | 89 | 0.121 | 1.3 |
| 6 | 8 | 1,741 | 838 | -0.036 | 1.3 | 777 | 0.035 | 1.0 | 126 | 0.123 | 2.2 |
| 6 | 9 | 2,007 | 971 | -0.013 | 1.7 | 894 | 0.014 | 1.3 | 142 | 0.129 | 2.6 |
| 6 | 10 | 2,152 | 1,044 | -0.035 | 4.7 | 958 | 0.011 | 1.3 | 150 | 0.189 | 7.8 |
| 6 | 11 | 2,176 | 1,058 | -0.026 | 6.5 | 967 | 0.011 | 1.3 | 151 | 0.183 | 7.9 |
| 7 | 8 | 1,203 | 580 | -0.038 | 0.8 | 532 | 0.011 | 0.1 | 91 | 0.031 | 0.1 |
| 7 | 9 | 1,469 | 713 | 0.000 | 1.5 | 649 | -0.016 | 0.7 | 107 | 0.055 | 0.3 |
| 7 | 10 | 1,614 | 786 | -0.032 | 4.1 | 713 | -0.017 | 0.7 | 115 | 0.142 | 5.0 |
| 7 | 11 | 1,638 | 800 | -0.018 | 6.1 | 722 | -0.017 | 0.7 | 116 | 0.131 | 5.1 |
| 8 | 9 | 684 | 338 | 0.061 | 1.3 | 293 | -0.046 | 0.6 | 53 | 0.036 | 0.1 |
| 8 | 10 | 829 | 411 | -0.013 | 3.3 | 357 | -0.033 | 0.7 | 61 | 0.186 | 3.7 |
| 8 | 11 | 853 | 425 | 0.012 | 5.4 | 366 | -0.032 | 0.7 | 62 | 0.162 | 3.9 |
| 9 | 10 | 411 | 206 | -0.124 | 3.1 | 181 | 0.023 | 0.1 | 24 | 0.370 | 3.2 |
| 9 | 11 | 435 | 220 | -0.060 | 4.9 | 190 | 0.022 | 0.1 | 25 | 0.280 | 3.4 |
| 10 | 11 | 169 | 87 | 0.191 | 3.1 | 73 | 0.004 | 0.0 | 9 | -0.274 | 0.6 |

**Figure S1.** Coefficients of variation (CV) among weekly tortoise movements and different sample sizes. CV was calculated from the distribution of bootstrapped means (with replacement) using observed distances in 19 tortoise-years containing at least 15 inter-burrow movements. Ranges of CV values within each movement sample are from these 19 tortoise-years. Boxes indicate interquartile ranges, whiskers ranges, and thick lines medians. The minimum (n = 15 movements) and maximum (*n* = 1 movements) CV are indicated with horizontal dashed lines. The median CV value at which 80% of the minimum was reached (*n* = 7 movements) is indicated with a thick dashed line.
